# Confabulation and Fluctuating Memory: A Case of Alzheimer’s Dementia in a Culturally Diverse Patient

**DOI:** 10.1002/alz.088516

**Published:** 2025-01-09

**Authors:** Nada Dahroug, Nidhal Siddig, Mayra Mezzoni, Nadir G Abdelrahman

**Affiliations:** ^1^ Michigan State University (MSU), East Lansing, MI USA; ^2^ Michigan State University, East Lansing, MI USA

## Abstract

**Background:**

Confabulation, the fabrication of details with short lucid intervals, hinders the diagnosis of Alzheimer’s and other dementias. This case report explores the complexities of diagnosing and managing Alzheimer’s dementia in a culturally diverse patient exhibiting fluctuating memory and confabulation, emphasizing challenges heightened by cultural and linguistic factors.

**Case:**

An 84‐year‐old African descent man resided with his family in the United States, independent in his basic daily activities but struggling with instrumental tasks due to memory impairment. While demonstrating clear recollection at times, closer examination revealed fabricated details and disorientation regarding his current environment. Confabulation in this trilingual patient (English, German, and Arabic) posed additional challenges, necessitating a closer look at his cultural background, language barriers, and family dynamics. The patient was born and raised in Africa. He relocated recently to join his family in the US. The family was involved in managing medication adherence and therapeutic interventions. A specialist evaluated the patient, and the Montreal Cognitive Assessment (MoCA) indicated a score of 12/30, signifying moderate cognitive impairment. The assessment considered language nuances, cultural influences, and familial support. Laboratory tests were grossly unremarkable. Donepezil and Vitamin B12 were prescribed to the patient. Family members were educated regarding the use of storytelling as a therapeutic approach.

**Result:**

Family involvement played a pivotal role in holistic care coordination. Despite prescribed medications, the family has not initiated treatment due to the risks of side effects. Melatonin was used as needed for sleep disturbances. Predominantly non‐pharmacological treatment approaches were implemented, including reorientation and redirection. Storytelling as a therapeutic approach was effective.

**Conclusion:**

This case underscores the significance of culturally sensitive approaches in cognitive assessment and holistic care coordination, emphasizing the need for continued research on Alzheimer’s presentation in diverse populations. The abstract serves as an invitation to discuss effective strategies for diagnosing and managing Alzheimer’s dementia in culturally diverse patients, promoting inclusivity and optimal care.

